# The commodification of mobile workers in Europe - a comparative perspective on capital and labour in Austria, the Netherlands and Sweden

**DOI:** 10.1186/s40878-017-0048-0

**Published:** 2017-02-14

**Authors:** Mark van Ostaijen, Ursula Reeger, Karin Zelano

**Affiliations:** 1 Department of Public Administration and Sociology, Erasmus University Rotterdam (EUR), PO Box 1738, Room T17-53, 3000 DR Rotterdam, The Netherlands; 2 Institute for Urban and Regional Research/Austrian Academy of Sciences, Postgasse 7/4/2, A-1010 Vienna, Austria; 3 Department of Political Science, University of Gothenburg, Box 711, 405 30 Gothenburg, Sweden

**Keywords:** Commodification, EU mobility, CEE migration, Labour market relationships, Ideal types

## Abstract

One of the defining features of contemporary Europe is the freedom of movement of persons. Despite its advantages, this ‘freedom of movement’ is also contested, since it has been shown to cause discrimination, exploitation and pave the way for a ‘race to the bottom’. How can we understand the social-economic consequences of free movement in Europe? To answer this question, we developed a typology along the dimensions *value of work* and *degree of power* which delivers four ideal types of labour relationships: exploitative, deprived, greedy and esteemed. This has been applied to Central and Eastern European (CEE) workers in Austria, the Netherlands and Sweden.

Our study shows *dual labour market strategies* of *both* capital and labour agents, using on the one hand strategies of cost minimisation, and on the other hand compliance strategies and dual frames of reference, both of which contribute to a low degree of freedom and a low value of work. It addresses the responsibility and significance of *both* capital and labour contributing to *exploitative* and *greedy* relationships throughout all three cases. The results contribute to a more balanced understanding of the responsibilities towards the ‘shadow sides’ of free movement in the EU, as it shows that not all free movement of persons is totally free. Moreover, instead of bold political statements, it demonstrates the relevance of a more differentiated perspective on the downsides and benefits of European free movement.

## Introduction

*‘[…] the new social realm transformed all modern communities into societies of laborers and jobholders’* (Arendt, 1958, p. 46)

The EU ‘enlargements’ or ‘accessions’ of 2004 and 2007 have come to shed a new light on the European project. New member states joined the EU, which led to large amounts of Central and Eastern European (e.g. Polish, Hungarian and Romanian) workers moving to West-European countries (such as Austria, Sweden and The Netherlands). Since CEE migrants have become one of the most significant categories of mobile EU citizens to arrive in the ‘old’ European member states, we primarily focus on ‘CEE’ (Central and Eastern European) mobile workers or ‘migrants’ in our analysis. Soon after the enlargements, European mobility rose to the top of the political and media agenda in France, Italy and Britain (Favell & Nebe, 2009). Academics have also shown the danger of discrimination and exclusion of EU labour within ‘a regime of exploitable and controllable labour’ (McDowell, Batnitzky, & Dyer, 2009; McKenzie & Forde, 2009; Skrivankova, 2010; Fox, Morşoanu, & Szilassy, 2014; McGauran, de Haan, Scheele, & Winsemius, 2016; Glick-Schiller, 2009, p. 124).

While these studies deliver rewarding empirical micro-level insights about the consequences of free movement, theoretical meso- or macro-level dynamics *between* employers and employees remains a somewhat underdeveloped topic. Or, as stated by Fudge and Strauss (2014), p. 3: *“While these processes […] have been explored individually, there has been relatively little work to date that has attempted to explore the linkages between them”.* This is surprising because – with the free movement of people as one of the key instruments to optimise the European ‘Single Market’ – it is important to understand the market relationships *between* employers and employees. To do so, we focus on this relationship in three member-states with a comparable and significant inflow of EU mobile citizens, yet with different institutional settings and varying transitory regimes: the Netherlands, Sweden and Austria. The main research question is: *‘What is the character of socio-economic relationships between capital and labour agents concerning the free movement of people in Austria, the Netherlands and Sweden?’* To answer this, we use data from a multiple stakeholder analysis (online surveys, interviews and focus groups) with respondents in the three countries.

The first part of this article includes a theoretical conceptualization of the *relationship* between capital and labour, resulting in a typology of *ideal-typical* relationships according to two dimensions: the *degree of power* and *the value of work*. The second part of the article presents the empirical findings and analyses. The final part reflects on the findings and relate to the debate considering the downsides and benefits of free movement of persons in Europe.

## The commodification of European mobile work

In contemporary studies, the vast body of literature on ‘renewed’ European East-west migration has often focused on analysing stocks and flows, on migration patterns, contributions to labour migration and integration (Guild & Mantu, 2011; Gabriel & Pellerin, 2008; Menz & Caviedes, 2010; Engbersen, Leerkes, Grabowska-Lusinska, Snel, & Burgers, 2013). With the change in the European legal-political framework and the increase of free movement, academic interest also shifted to post-accession migration, addressing issues of labour market segmentation and de-qualification (Black, Engbersen, Okolski, & Pantiru, 2010, Glorius, Grabowska-Lusinska, & Rindoks, 2013; McGauran, de Haan, Scheele, & Winsemius 2016). There is a growing awareness among migration scholars that although EU-internal migrants have the same rights as citizens in the receiving EU countries, they may still face significant barriers and have ‘integration needs’ similar to migrants from outside of the EU (Collett, 2013; Reeger, forthcoming). These needs relate mostly to language skills and access to information, especially concerning the host country labour market (Ciupijus, 2011; Reeger & Enengel, 2015). The perception and continued description of EU migrants as particularly hardworking and their use as such also hinders their social participation and language learning, as seen for example in the British context (McKenzie & Forde, 2009). But is this really the case? Since we have various studies on single cases and on the position of employees or employers in these cases, we study the meso-perspective between employers and employees in several European countries from a comparative and more inter-relational perspective.

The free movement of people is regarded as an important component in *‘creating a European employment market’* (Eurobarometer, 2004; Heinz & Ward-Warmedinger, 2006, p. 7), whereby EU citizens are obliged to have comprehensive resources at their disposal so as not burden the welfare state system of their host countries. Having a working relationship or contract (with a company, an employer or with themselves as self-employed) is therefore a defining element of their legal status (Rosewarne, 2010; Ruhs & Anderson, 2010; Snel, Faber, & Engbersen, 2015). Consequently, mobile *citizens* are *de facto* mobile *workers* on a mobile *market*. Therefore, we approach European freedom of movement primarily as a market phenomenon. One of the most defining characteristics of a liberal market is the *commodification of labour* 1 (Polanyi, 2001; Shields & Grant, 2010, p. 61),^2^ a situation where work is freely bought and sold as ‘labour’ on a market at a market price, such as ‘wages’ (Polanyi, 1977, p. 13). Consequentially, through market mechanisms, labourers can (and will) be transformed into tradable commodities (Polanyi, 2001; Marx, 1978/2010).^3^ We take labour commodification as a conceptual starting point in understanding European free movement. In contrast to other studies, however, we conceptualise labour commodification as a *relational* rather *problematizing* concept to understand market relationships (Papadopoulos, 2005; Shields & Grant, 2010; Rosewarne, 2010).^4^ In other words, labour commodification is not considered inherently problematic or negative in a normative sense, but as a *relational concept* for understanding the relationship between capital and labour agents in the liberal market of the EU (Anderson, 2010). To study these *relations* between market actors, we include two elements in the analysis: the *degree of power* and the *value of work*. In the following, we explain how both elements are operationalised.

## Labour commodification as a relational process

*‘Immigration can be seen as a labor-supply system particularly suited to the needs of firms where the organisation of the labor process entails low wages and powerless labor’* (Sassen, 1988, p. 40).

### Degree of power

Some scholars have argued that labour power is a mere fiction and that employers do not buy labour power, but rather ‘the power to command’ (Anderson, 2000). For workers to counterweigh this, a defining element is to what extent the individual worker holds *agency*, understood as *‘exerting some degree of control over the social relations in which one is enmeshed, which in turn, implies the ability to transform these social relations to some degree’* (Sewell, 1992, p. 20). In light of this, we adhere to a gradual definition of the *degree of (worker) power* as a ‘continuum’ of (un)freedom (Ruhs & Anderson, 2010; Fudge & Strauss, 2014).

Furthermore, labour agency can be restricted by institutional, cultural or regulatory constraints to make independent decisions (Fitzgerald, 2007; Holgate, 2005; Favell & Nebe, 2009; Fox, Morşoanu, & Szilassy, 2014). Conversely, when constraints are limited and dependencies are legally separated and regulated, this could stimulate labour agency. It can also be stimulated by transparent, accessible and available information about rights and duties vis-à-vis employers.^5^ This applies to self-employed persons as well as other employees, especially those who are active in service-oriented professions (e.g. the IT sector, engineering or the arts) (Salt, 2008; Kelo & Wachter, 2004). The importance of information for the power relation between employers and employees has been shown by several other studies, showing trade union membership as counterweighing corporate interests (Berntsen, 2015). Studies have even shown that in some sectors workers are selected when they ‘lack power within the labour market’ since ‘*it is also their powerlessness which makes them profitable’* (Sassen, 1988, p. 40; McKenzie & Forde, 2009, p. 155; Piore, 1979). This powerlessness can be emphasised by multiple dependencies, including access to medical care, transport and housing (Van Ostaijen, Faber, Engbersen, & Scholten, 2015). Therefore, to study the degree of power, we analyse *individual* and *corporate* investment and compliance *strategies* related to the labour process and working conditions. Generally, this degree of power *‘’in which this new arrangement of labour takes place –and its human costs- are all too rarely addressed within migration studies”* (Glick-Schiller, 2009, p. 125).

### Value of work

Studying European labour mobility cannot solely be done based on the *degree of power.* Rather, it should also incorporate how capital and labour agents *value* work. Although value of work can be defined rather broadly, this study primarily defines it as wages next to work conditions.

This primary focus on wages is not coincidental. ‘Wage improvement’ is one of the most commonly mentioned indicators of why EU citizens move, for instance to the Netherlands (Engbersen, Snel, Ilies, Leerkes, & Van der Meij, 2011). However, European movement sometimes comes at the price of low wage jobs, job insecurity and marginal positions on the labour market (Favell & Nebe, 2009; Janta, Ladkin, Brown, & Lugosi, 2011; McDowell, Batnitzky, & Dyer, 2009; Ciupijus, 2011; Fox, Morşoanu, & Szilassy, 2014), or as Favell expressed it: ‘*Ambitious “new Europeans” are in danger of becoming a new Victorian servant class’* (Favell, 2008, p. 711). Some studies show how employers, in their competitive *corporate strategies* for cost minimisation, strategically use loopholes and opacities to reduce labour costs by recruiting mobile workers (Fellini, Ferro, & Fullin, 2007; Houwerzijl, 2014; Berntsen, 2015). Such cost minimisation strategies limit the valuation of work, in terms of suppressed wage levels (Ruhs & Anderson, 2010). However, this does not always have to be the case since wages can still be high or in accordance with national norms. This is mostly the case for knowledge workers, highly skilled expats and creative professionals (Iredale, 1999; 2001). Nevertheless, being highly educated or highly skilled does not safeguard a person from the undervaluation of work. Studies also show that much talent and human capital is wasted due to ‘downward mobility’, ‘undervaluation’ and problems of de-qualification (Kelo & Wachter, 2004; Favell & Nebe, 2009).

Next to corporate valuation, we focus on the individual valuation of work. This cannot be done by adopting a fixed approach to wages, since studies have shown that mobile employees sometimes accept a lower socio-economic position in the short run as an investment for their career in the long run (Ruhs & Anderson, 2010; Pietka, Clark, & Canton, 2013). Such compliance or coping mechanisms can become part of tactics to increase income and save on household expenditure (Datta et al., 2007; Holgate, 2013). An initial acceptance of low-paid jobs is found in both low-skilled and high-skilled work (Iredale, 1999; 2001; Voitchovsky, 2014; Glorius et al., 2013; Lillie & Greer, 2007; Berntsen & Lillie, 2012). This highlights the importance of *time* as a factor in the valuation of work by mobile employees. As time passes and social contacts, language skills and information resources increase, the willingness to comply decreases (Piore, 1979; Anderson, 2010; Datta et al., 2007). For example, Dutch research indicates that almost 40% of the Bulgarian population in the Netherlands complied with lower wages than the legal standard of minimum wages (Engbersen et al., 2011, p. 44). Comparing their situation with the Dutch standard but also with the situation of peers in the country of origin, this is known as a ‘dual frame of reference’ when immigrants compare their situation with their country of origin (Suarez-Orozco, 1987; Waldinger & Lichter, 2003). This makes wage valuation an ambiguous and complex element in the individual valuation of work, and shows the importance of taking time and contextual factors into account. Table 1 offers a concise overview of the elements and indicators that guide our study.

**Table 1:** Operationalisation of labour commodification

Degree of power		
* Elements*	*Defined as*	*Indicators*
Individual strategies	Investment or compliance strategies for labour agency	Valuation of labour agency (Independency and autonomy in work decisions, information, trade union membership, voice)
Corporate strategies	The use of resources to invest in labour agency	Fulfilment and valuation of agency conditions (Autonomy in work floor decisions, accessibility of information, trade union membership)
Value of work		
* Elements*	*Defined as*	*Indicators*
Individual strategies	Investment or compliance strategies for labour activity	Valuation of primary and secondary labour conditions (wages, information, contractual and collective agreements)
Corporate strategies	The usage of resources for development or investment in labour	Fulfilment and valuation of primary and secondary labour conditions (wages, information, contractual and collective agreements)

**Fig. 1:**
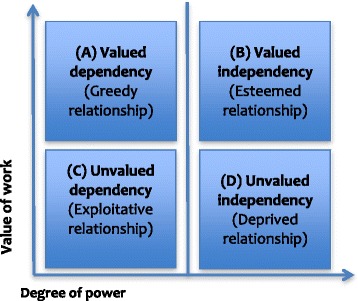
Typology of labour-capital relationships. Holds mainly descriptive and explorative value. It does not assume a causal relationship between both axes, but based on existing literature, we combined insights to formulate expectations. Source: own design

This conceptualisation of *labour commodification* enables a sketch of four ideal types, understood as *“constructed concepts endowed with a degree of consistency, seldom found in actual history”* (Weber, 2002, p. 55). Ideal types draw attention to specific features of a phenomenon, in order to build a picture of its key characteristics to reveal and explain social phenomena. Combining these two dimensions, ‘value of work’ (weak and strong) and ‘degree of power’ (weak and strong) result in four ideal-types of labour-capital relationships which are visualised in Fig. 1 in four quadrants:

1. *Unvalued dependency* (low value/low power) combines a low exchange value with a low degree of power. This type is termed ‘exploitative relationship’;
2. *Unvalued independency* (low value/high power) combines a low exchange value with a high degree of power. This type is termed ‘deprived relationship’;
3. *Valued dependency* (high value/low power) is a combination of a high exchange value of work with a low degree of power. This type is termed ‘greedy relationship’;
4. *Valued independency* (high value/high power) is a combination of a high exchange value of work with a high degree of power. This type is termed efficacious or ‘esteemed relationship’.

We expect low-skilled workers to be mostly in *exploitative relationships* because of low work valuation (wages) and a low autonomy on work-related decisions, which decreases employee agency (Expectation 1). We expect low-skilled workers contracted by temporary employment agencies mostly in *greedy relationships* because their wages regularly meet economic and legal standards, but because of clustered contracts, they depend on their temporary employment agency in a range of social domains such as housing, social security and transport services (Expectation 2). Based on the work of Coser (1974), ‘greediness’ indicates when organisations demand ‘total dedication’ of their members and try to ‘limit the demands of competing roles and status positions’. When the value of work is seen as convenient but when personal and professional freedoms are minimal, we typify this as a greedy relationship. Subsequently, we expect self-employed persons to be in *deprived relationships,* because they have formally a high degree of power or freedom to commodify their work, but because of their individuality, they largely do not profit from collective agreements, which could lead to underpayment or self-exploitation (Expectation 3) (Reeger & Enengel, 2015; Berntsen, 2015). Finally, we expect highly skilled workers to be in *esteemed relationships* because their labour potential is mostly valued according to legal standards (by wages and secondary conditions) and they maintain a relatively high degree of power, independence and autonomy in their labour market position (Expectation 4) (Florida, 2002). For this expectation to be met, it is important that highly skilled workers have highly skilled positions, since alternative studies also show the undervaluation of highly skilled persons in low skilled positions (Trevena, 2013; Reeger & Enengel, 2015). Overall, we expect that low-skilled mobile workers will most likely cover the left rectangle of Fig. 1 (quadrant A, C or D) while highly skilled mobile workers most likely cover the right rectangle of Fig. 1 (quadrants A, B and D) (Jordan & Duvell, 2002). We will now apply these ideal types to our empirical case studies. For that purpose, we completed a multiple stakeholder analysis, introduced below.

## Methods

This study involves an in-depth qualitative case study of labour-capital relationships within the European freedom of movement, specifically focusing on East–west labour movements.^6^ Intra-EU mobility was selected as a strategic case for the understanding of labour commodification due to its distinct liberal-economic objectives and its market-led character.

To answer our research question we studied stakeholder assessments in three countries that had taken part in the IMAGINATION research project.^7^ ‘Stakeholders’ are defined as actors who deal professionally with European mobility, selected on the basis of their professional affiliation, and distributed across public, private and NGO sector. These stakeholders were professionals working at temporary labour agencies, employment services, chambers of commerce, employers’ organisations, workers’ organisations, civil society migrants’ organisations and civil servants on the local and national level. Table 4 in Appendix shows an anonymised list of respondents used for quotations in this article. By studying the assessments of professionals dealing with ‘mobile work’ in a broader sense, we were able to trespass direct tense or conflicting interests between employers versus employees. In other words, we did not interview employers or EU workers active on the labour market, but instead, approached professionals whose work relates to ‘mobile workers’, who are or have been a ‘mobile worker’ in the past, or are otherwise professionally affiliated to issues regarding ‘mobile work’. The professionals are therefore able to reflect on *relational* elements from a distant and more reflective perspective.

This multiple stakeholder analysis was designed as part of a stepwise research methodology, including an online survey, semi-structured interviews and focus groups. The online survey was carried out in spring of 2014 and resulted in 129 returned questionnaires in the three countries (see Table 2), a response rate of about 80%.^8^ The survey served as a mapping exercise, establishing a primary inventory of relevant implications of CEE mobility. The questionnaire began with the stakeholders’ concrete work, tasks and CEE clientele, followed by a core section investigating implications in different policy domains (labour market, housing etc.). In a closing section of the survey, the stakeholders were asked to elaborate on issues they considered particularly relevant with regards to CEE mobility. Initially we had an equal distribution of respondents per professional domain (public, private, NGO). The online survey was followed by a series of 52 interviews with stakeholders. Almost all the interviews were conducted during summer/autumn of 2014. Respondents were asked about what they professionally perceived as the implications of intra-EU mobility, in four main domains: the labour market; housing and neighbourhood; registration, social security and participation; language and education. Comparing these four domains, labour market implications were most prominent, delivering in-depth insights about the relationship between employers and employees. For the selection of respondents, we used the same professional affiliation criteria as the online survey. Following on from the survey and interviews, we conducted focus groups in which participants were again selected based on their professional affiliation. Sometimes respondents were asked twice to participate, since the aim of the focus group was to gain new knowledge, but also deepen the understanding of and corroborate the results from the survey and interview findings. Table 2 summarises the number of respondents in all cases.

**Table 2:** Numbers of respondents in the three stages of data gathering

	Urban region	Online survey	Interviews	Urban Living Lab
Austria	Linz	N = 23	N = 8 (9)	N = 8
	Vienna	N = 23	N = 5 (7)	
	National level	--	N = 1	
The Netherlands	The Hague	N = 15	N = 5	N = 16
	Rotterdam	N = 15	N = 5	
	National level	N = 16	N = 2	
Sweden	Gothenburg	N = 22	N = 8 (12)	N = 30
	Stockholm	N = 15	N = 5 (7)	
	National level	--	N = 4	

Source: Reeger & Enengel, 2015

All focus groups and interviews were recorded, transcribed and subject to qualitative document analysis after approval by the participating stakeholders. This was all part of comparative data analysis grids, exchanged within the project IMAGINATION. The analysis of the data was based on theoretical and empirical confrontation, taking an abductive approach: Positioned between inductive and deductive reasoning (Timmermans & Tavory, 2012). By back-and-forth reasoning (Berg & Lune, 2004), the data were analysed by the grid which all had to relate to the four identified domains. To increase reliability, several rounds of interpretations were undertaken and discussed in the research groups and between the researchers.

Finally, it is important to mention the biases in the research design. A multiple stakeholder approach implies a problem-oriented perspective because the professionals’ daily work is focused on dealing with issues and problems related to CEE migration. We also acknowledge that by interviewing professionals about ‘implications’, most respondents will focus on the most ‘visible’ and ‘pressing’ implications. In other words, interviewing professionals about ‘implications’ has a risk of overemphasising ‘problems’ within specific sectors. While we acknowledge this as a bias in our research design, we aim to compensate that effect using data triangulation.

## Background: EU mobility in The Netherlands, Sweden and Austria

**Fig. 2:**
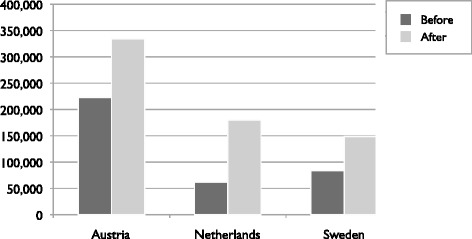
Number of CEE residents in Austria, the Netherlands and Sweden before and after EU-enlargements of 2004 and 2007. Source: Sert, 2014

The Austrian, Dutch and Swedish cases were selected as *revelatory* case studies (Yin, 1996) as these countries experienced a relatively significant increase in mobile workers through the EU-enlargements of 2004 and 2007 (see Fig. 2). Next to this, all three countries had different transitory regimes in opening their labour markets for European workers: Sweden was among the first European member-states opening their labour market for A8 countries in 2004, the Netherlands followed in 2007, while Austria extended the transitional arrangements until 2011.

The case studies also feature diversity in their political-institutional structure and socio-economic outlook, shown in Table 3.

**Table 3:** Political-institutional outlook case studies

Country	Austria	Netherlands	Sweden
Politico-administrative model	Federal	Unitary decentralized	Unitary
State - Social partners relation	Corporatist	Semi Corporatist	Non Corporatist
Type of Welfare State	Conservative	Mixed	Social Democratic
Opening of the labour market (A8)	2011	2007	2004

Source: Zelano, Bucken-Knapp, Hinnfors, & Spehar, 2016

These variances guided the comparative case study approach. We will now proceed with a concise overview of each case study country.

### The Dutch case

Historically, the Netherlands played a founding role in the establishment of the EU and the euro and has been a proponent of the European free movement regulations (Hollander, 2013). Migration from Central and Eastern Europe (CEE) to the Netherlands did not begin with the EU-enlargements of 2004 and 2007, the number of (officially registered) residents from CEE countries did however increase rapidly. In the late 1990s, there were about 50,000 CEE residents in the Netherlands, in 2003, shortly before the EU enlargement of 2004, this number had grown to 62,000, adding up to 120,000 CEE residents (Statistics Netherlands, 2012). Previous research on the labour market position of this population, indicated that a large majority of recently arrived CEE migrants in the Netherlands had (employed or self-employed) work (Dagevos, J. (red.), 2011; Gijsberts & Lubbers, 2013). Others found that this work was mostly based on flexible employment relations, or even on an informal basis (Engbersen et al., 2011). Most studies found that CEE migrants are generally concentrated in elementary occupations, particularly in the Dutch horticulture, but also in construction, cleaning, catering and in private households (Dagevos, J. (red.), 2011; Gijsberts & Lubbers, 2013).^9^ Engbersen et al. (2011, 2013) indicate that the majority of their respondents (62%) were either unskilled manual or agricultural workers. However, they also found that some CEE migrants were working in ‘higher service’ occupations. These studies showed that CEE migrants are a more differentiated group than often expected, including low, medium and highly skilled migrants, while this last category more limited.

One of the most important labour market specificities in the Dutch case is the significant role of temporary employment agencies, as one of the main providers of mobile work (Fellini et al., 2007; Brinkmeijer, 2011). The large agricultural and horticultural sector in the southern and western parts of the Netherlands see these intermediaries play a pivotal role in the brokerage of capital and labour. Consequentially, this large sectoral demand creates a substantial amount of low-skilled mobile work in the Netherlands (Engbersen et al., 2011, 2013; Gijsberts & Lubbers, 2013), an important specificity regarding the Dutch case.

### The Swedish case

Sweden joined the EU in 1995, and after the EU enlargements in 2004 and 2007, Sweden (alongside Ireland and the UK) decided to open-up the labour market to new EU citizens entirely and abstain from any transitional arrangements. Very soon after this opening of the labour market, a conflict arose that still characterises discussions about the Sweden-EU relationship. A company hired Latvian workers to construct a school in the municipality of Vaxholm, without collective agreement. After national court proceedings, the Court of Justice of the European Union (ECJ) finally ruled that the blockade represented a restriction on freedom to provide services under Article 49EC. The series of events, known as ‘the Vaxholm case’ or simply ‘Laval’, has become a strong national narrative, and serves as a metaphor of how the ‘Swedish model’, founded on independent negotiations between labour and business, is challenged by the free movement of labour. The work conditions of low-skilled, manual labour in general and of posted workers specifically, have since been a constant factor in the political debate.

CEE migrants can be found in all sectors of the Swedish labour market, but predominantly in construction, forestry and the sector for private household services (mostly cleaning). Employment relations in these sectors are mixed, contracts range from formal and semi-formal to purely informal ones. Estimations of the ‘true’ population of posted workers and informal workers in Sweden vary significantly between employer and employee organisations. According to the official register of posted workers, approximately 40% of posted workers during the first half of 2014 came from the CEE region, especially Poland (Swedish Work Environment Authority, 2014).

### The Austrian case

Austria joined the EU in the same year as Sweden, in 1995, after a referendum in 1994 with 67% of eligible voters being ‘pro EU’. As to transitional provisions, Austria lobbied along with the Netherlands and Germany for implementing these restrictions for as long as possible in order to protect its labour market (Kraler, 2011, p. 37). The transitional arrangements were finally abolished in 2011 and 2013. At the beginning of 2016, more than 360,000 CEE citizens were registered in Austria, out of which Romania (82,971) and Croatia (70,255), Hungary (63,608) and Poland (57,604) were the most common countries of origin. In total, the share of CEE citizens in the population amounts to 4.2% (Fassmann, Kohlbacher, & Reeger, 2014).

CEE migrants can be found in all parts of the Austrian economy and in all kinds of positions. The longer duration of the transitional provisions resulted in an above average share of self-employed CEE citizens, as this was one of few viable ways to work in Austria during the transition period. Still, these self-employed are often manual workers in positions that do not match their qualifications compared to non-migrants. It has also been shown, that the longer CEE migrants stay, the more likely they are to be able to obtain jobs matching their qualifications as they improve their language skills, widen their networks and improve their knowledge of the Austrian labour market (Fassmann, Kohlbacher, & Reeger, 1995, p. 38).

One factor that marks Austria out a special case compared to the Netherlands and Sweden is its spatial proximity to the CEE region. All the numbers given so far refer to officially registered CEE migrants and thus provide only a part of the bigger picture. For people working in Austria and living ‘at home’ in e.g. Slovakia or Hungary, there is no need to register and they are thus not included in population statistics. There are, e.g. about 50,000 women from CEE active in care work in private households, going back and forth on a biweekly or monthly basis. Spatial proximity thus brings along forms of mobility that are not feasible in the Netherlands or Sweden.

We will now turn to a discussion of the results on labour-capital relationships in the case study countries. We present the results along the lines of the analytical grid [corporate and individual strategies], and not case-to-case, to offer a comparative perspective.

## Results

### Corporate strategies

First of all, especially regarding low-skilled work, Austrian, Dutch and Swedish respondents indicated that employers actively search for the cheapest and easiest labour options. In all national settings, employers need to pay a legally defined minimum wage, depending on a worker’s age and specific area of work. But even with such legal standards, wage variation occurs, described here by a representative of a temporary employment agency in the Netherlands:

*‘There are large wage differences between native and mobile workers. Every person encounters that, sooner or later, they will see that they get lower wages for the same work than Dutch people, which has been the case for years now’* (Matti).

Dutch respondents confirmed that this is also one of the main reasons employers actually prefer mobile workers, as stated by a representative of a temporary employment agency, they are ‘*cheaper and more profitable’* (Jonas). The Swedish local ombudsmen in Stockholm and Gothenburg also indicated that CEE workers work longer hours, for less money and are exposed to more risk than Swedish workers, as one representative of the Workers Union in Sweden describes:

*‘These construction corporations blame each other for having cheap labour force on their constructions. All the time, the focus is to keep the expenses low and they know very well what they are doing. They know that they can pay these workers a lot less than native Swedes and thus earn a lot of money using underhand means* (Dennis).

The contracts are not necessarily informal, but range from purely informal to semi-formal ones. According to Swedish trade union representatives in the construction sector, free movement has blurred the distinction between formal and informal labour contracts, as perfectly legal employment arrangements can coexist alongside more dubious setups on the same construction site. This creates a complex picture of long contract chains and a blurry distribution of responsibilities between contractors. Again, the representative of the Workers Union in Sweden talks about this complexity:

*‘The workers are getting the right salary, but it is not a question of salary, it is a question of taxes. (…) The corporations are not paying the right taxes. It is the same if you work for a Swedish employer, and you get your net payment, but not your payslip. And if you do receive your payslip, the taxes are not specified on it. When you 1 day declare your taxes, the Tax Agency is wondering why you have not paid your taxes - and you do not have a payslip to show how much you owe. Then it is up to you to pay what you are taxable, that should have been paid by your employer’* (Dennis)

Such tax evasion strategies make it possible for some employers to pay less than they should. There findings can be seen reflected in all three national samples, dominated by the perception that employers are first and foremost – and not only with regard to CEE workers – interested in cheap labour. As one representative of the Austrian Public Employment Service indicates:

*‘The goal is to find good personnel for as little money as possible, the best and least complicated persons’* (Martin).

Cost-minimisation strategies cause employers to avoid searching for native workers, as they need to pay higher wages to them than they do for mobile (foreign) workers. At the same time, premiums and taxes on mobile worker pay are not always imposed as they should be. This tax discrimination generates a nationality based approach, also indicated by Dutch employers, which leads to ‘*group-to-group’ thinking*, where employers select workers on the basis of their national background (see: McKenzie & Forde, 2009). In Austria too, one respondent indicated that: *‘It happens frequently, that employees with a migrant background doing the same job get less salary than Austrian co-workers. There are skilled workers getting the same amount of money as low-skilled workers. A clear case of discrimination by the employer’* (Paul)*.* Similarly, trade union representatives in Sweden gave voice to a similar nationality-based focus of employers, that is eventually mirrored in the organisation of labour:

*‘There is an increasing practice to mix Swedish and EU labour. It doesn’t work. In the end the Swedes are out of work. They train them, and once they are trained, they force out the Swedish labour. The Swedes themselves have been very stupid. Because they have let other people do their work. Alternatively, if you allow that, these people should have done so according to collective agreements’* (Andreas)

This nationality-based approach is not only guided by economic incentives, but also by the information resources of workers. As stated by one Dutch respondent of a temporary labour agency: if one group ‘knows enough’ about their alternatives and opportunities, employers likely switch to another group:

‘*Two or 3 years ago I was really busy with selling Polish personnel well. […] Now, we have a bit the same problem as back then. I hear from my customers: Those Polish people are a bit too experienced with the work here, the conditions, rules… maybe it is time for a new Polish person!’* (Matti).^10^

Having *less* knowledge of rights, duties, legal status and labour market position can perversely result in a *higher* valuation by the employer, while at the same time weakening the power of the individual worker. As an example, some Dutch employers value the lack of Dutch language skills, because knowing Dutch can be an indicator for ‘someone who knows too much’. As stated by a representative of a temporary employment agency:

‘*Most of those people speak the language. Previously, that was an advantage. Now this is for some customers (*companies hiring temporary personnel, MvO*) a disadvantage. Because with the knowledge of language they begin to talk too much and too fast with other people, comparing salaries. And they are not busy with their work. But only with living, let’s say it like that.’*(Matti).

Instead of investing in human capital or making information transparently accessible, this indicates that employers experience a range of incentives to limit resources of information. This emerges as a corporate strategy to minimise resources invested in employee development [value of work]. The educational background, qualifications, independency or autonomy are not always rewarded to meet working aims. Austrian stakeholders also indicated that insufficient information was provided, especially by intermediaries in the countries of origin:

‘*Experiences with these agencies are rather bad. They do not inform the care workers properly prior to their work in Austria, they consciously don’t, because they make a lot of money from this disinformation’* (Barbara).

This shows similarities with the Dutch situation; Dutch temporary employment agencies officially need to provide Dutch employees with information booklets about work, rights, duties and information about their collective agreement. However, this is not met in practice, as one representative of a temporary labour agency (Joachim) stated *‘in the end, it is the responsibility of the individual worker*’.

Furthermore, some employers indicated that they add ‘services’ (such as insurance contracts, travel and housing accomodations) to the labour contract. For example, one Dutch temporary employment agency stated that they have *‘700 bikes’* to reduce transportation costs. In this way, some Dutch employment agencies hold an *umbrella approach* with *all-inclusive practices* like a representative of Dutch temporary employment agencies indicated:

‘*Yes, it was just a package-deal […]. So, recruitment, selection, transport, housing, labour, labour-housing transport, this was all within that package. And what we see now is that all the different parts, it becomes more a model of choice. […] This makes it a bit unmanageable. And also a bit less comprehensible*’ (Joachim).

Such temporary employment agencies cluster different services into one comprehensive labour contract as a corporate strategy of cost minimisation. This comprehensive approach is mostly framed as a *service oriented* model, but when ‘housing’ or other services become a labour condition, this limits the possibilities for employees to *‘vote with their feet’* (Joachim) and it increases the possibility for employment agencies ‘*to do something wrong*’ (Matti*)* as two representatives of employment agencies indicated [degree of freedom]*.* Next to the Dutch examples, Swedish stakeholders reported that dependency occurs when the landlord and the employer are the same person. If employees lose their job, they also become homeless. Some Dutch employers mentioned the ‘manipulative’ character when housing is part of the labour contract of employees, causing ‘double dependencies’ and making workers more vulnerable, decreasing employee agency and locking the employee into a more subordinate position [degree of freedom]. As a result, employees are afraid to speak up, as they risk repercussions such as losing their job, their house and their social network. In summary: Wages, contracts and collective agreements are managed within a European framework and equal to all European mobile workers in theory. However, in practice, stimulated by profit-maximising and cost-minimising strategies of employers, our data indicate unequal treatment of mobile workers based on their national background, legal status and how well-informed they are. Oftentimes, these three aspects are mutually reinforcing, perpetuating the vulnerable position of workers in relation to their employers.

### Individual strategies

In this section, we move on to the individual strategies that highlight employer-employee relations. In our data, Dutch respondents indicated that some workers accept (temporary) jobs which do not match their educational profile. This points to individual compliance strategies and investment tactics to confine their educational profile in the short run to invest in their socio-economic profile in the long run. Over time, this could cause ‘dequalification’, ‘deskilling’ or the ‘undervaluation’ of human capital. In Austria, employers are aware that they recruit staff with higher or sometimes simply other qualifications than required, benefiting without actually compensating:

*‘Yet people come who are better qualified and take up jobs where they earn more than at home. So highly qualified people take up low-qualified jobs like sitting at supermarket checkouts. Still they earn much more and often know German well … For those who come from Slovakia and Hungary, low-wage jobs are in fact high-wage jobs’* (Martin).

By accepting a more subordinate position, mobile workers compromise autonomy, voice and independence in the short run, aspiring for improvement in the near future [degree of freedom]. Dequalification develops out of a lack of information about the recognition of qualifications, combined with weak networks, insufficient language skills and a low demand in certain high-skilled professions, contributing to their vulnerability. This highlights the significance of accessible and high quality information as well as the ability to act on it, or ‘to speak up’. Multiple Dutch respondents indicated that mobile workers are only nominally aware of their rights and have limited capabilities to gain additional information, especially those with a low socio-economic status [value of work & degree of freedom] because of their ambition to improve their wages. But as stated by stakeholders in Austria, mobile workers do not have to rely only on the information made available by employers, but can also look for themselves:

‘*Migrant communities often compensate for the lack of information, also due to a language barrier. Sometimes they provide useful information, sometimes they put false rumours in the world. But nobody really considers themselves to be responsible*’ (Daniel).

Swedish stakeholders also indicated the limited access to information of mobile workers. The trade union in Gothenburg reported a general lack of knowledge amongst mobile workers about their rights. Furthermore, they noted that initial contract agreements were to a large extent not met. Once in Sweden, workers have little choice but to work for inferior conditions than initially agreed on. Therefore, varied reasons lie behind the limited informational position of mobile workers, which in the end causes *informational asymmetries*, limiting individual strategies to develop autonomy, agency and independency [degree of freedom].

As such, mobile workers have – in theory – the opportunity to increase their skill level and to boost their social capital, language proficiency and professional portfolio, or as one Dutch former labour migrant indicated that especially *‘for highly educated people, labour migration is only for the experience, broadening your horizon. Wage is not the most important factor anymore’*. The value of work perceived by mobile workers is not only seen by improvements in terms of wages, but also in terms of secondary labour conditions, gaining tacit skills, knowledge and social capital that will strengthen their international profile [value of work]. Indeed, some are aware of their own ‘value’, indicated by one representative of the Workers Union in Sweden:

*‘The construction corporations are recruiting workforce in their home countries and most of them are very skilled craftsmen. The Poles have a higher income salary and they stick together and also stay longer than those workforces that are coming in smaller groups. The Poles know their own value.’* (Dennis)

Most workers value their work in varied ways. However, if we look at individual cases, we see that the situation of mobile workers in most cases does not live up to these ideal standards. Mobile workers want to improve their position and the future of their children in terms of socio-economic status, education and care. Or, as one former migrant worker that now works for a temporary employment agency indicated by a representative of a Dutch temporary employment agency:

*‘If I can earn 200 euro for stupid work in Poland, I’d rather prefer to earn 1000 Euro for stupid work in the Netherlands’* (Matti).

To improve their situation, there seems to be a broad willingness to comply with low wages, under the minimum wage level, which shows the significance of dual frames of reference. Moreover, compliance and investment strategies are not only applied to wages only (as saving tactic) but also more broadly to their overall social-economic and social-cultural status.

## Discussion

Before presenting our conclusions, we would like to highlight the specific limitations of this study. First of all, one main limitation of our data is that most respondents referred to low-skilled work, when they were asked about ‘implications of free movement’. This can be explained by the professional affiliations of our respondents (organisations working with temporary, low-skilled and informal workers, amongst others) but as expected earlier, most respondents focused on the most ‘visible’ and ‘pressing’ implications, which are mostly issues affecting the low-skilled sector. Data triangulation did not overcome this overemphasis, since in all data sources most attention was focused on the labour market domain in general and the low skilled sector of ‘manual workers’ in particular. This overemphasis led to a bias in our results which we will address.

Secondly, by carrying out a multi-stakeholder analysis we did not directly interview employers and employees. This enabled us to include more reflective positions, but it holds limitations to observe ‘real’ relationships in the workplace. It would have been interesting to conduct a large-scale survey and study the degree of freedom and value of work perceived by employers and employees on a larger scale and using a more direct approach. Future research could apply this framework to study the free movement of persons in such a direct relational perspective. Our selections give room for future research to broaden the research design. As some results reflect findings in other countries (such as Germany, Finland and the UK, see: Lillie & Greer, 2007), comparative studies could examine what role a different labour market context has on different commodified relationships. Such research could contribute to our understanding of free movement from a more differentiated perspective, including both the downsides and benefits of an even ‘freer’ Europe.

## Conclusions

Let us now reflect on our theoretical expectations and our initial typology of capital-labour relationships. First, all our cases show low or mediocre exchange value in the *value of work*. Focusing on valuation in terms of wages, this is below or in line with the minimum wage standard, while it is not strongly problematized by both (labour and capital) agents because of *strategies of cost minimisation* (of employers) on the one hand and *dual frames of reference* and *investment strategies* (of employees) on the other. Due to *compliance strategies* of individual workers affecting how they implement their professional and personal competencies and the *dual frames of reference*, mobile workers accept downward rewarding since they compare their situation not with Dutch/Swedes/Austrians but with their peers in their country of origin.

Second, we found low degrees of freedom (autonomy, agency and independency) in the cases studied. Employers experience, through strategies of cost minimisation, a range of incentives to limit value autonomy. Driven by competitive aims, employers value minimal knowledge and limit language investments. Our data even show employers’ incentives to keep employees as long as possible ‘on the sidelines’, by not investing in their socio-economic or social-cultural position. Such incentives are a burden for employee agency. While this is mainly unrewarded it is also low-problematized by *both* labour and capital agents because of the *competitive aims* (of employers) on the one side and *compliance strategies* (of employees) on the other. Individual workers comply, hoping to invest in future improvement. Therefore, the *freedom of work* is limited in valued by both labour and capital agents.

This adds up to the point that labour-capital relations in all cases are characterised by a low value of work and a low degree of freedom, adding up to *exploitative* or *greedy relationships*. It shows a very specific part of the typology, displaying that not all free movement of persons is totally free (Ciupijus, 2011). This confirms our first and second expectation. We cannot confirm our theoretical expectations three and four, because we did not come across *esteemed relationships,* and evidence for *deprived relationships* was also limited. This might be related to the bias in our research design as regards the overemphasis of our respondents on the low-skilled sector. However, utilising the typology was not done with the ambition of empirically overarching all possible types, but instead it was a heuristic tool, to conceptually approach the empirical data. Now that we have studied the data, we see an overemphasis in *esteemed and deprived relationships,* which can be related to our research design. However, it also of significance that in all three countries, most respondents refer to the low-skilled sector and define free movement in these terms. By acknowledging the limitations of our study, we do not claim that the results can be used to generalise about the complete labour markets of Austria, Sweden or the Netherlands, or that they are applicable to all segments of the European labour market. However, we do think that by focusing in on the specificities of *esteemed and deprived relationships,* we do see some interesting similarities which could be an incentive for scholarly and policy attention.

More generally, our study shows *dual labour market strategies* of *both* capital and labour agents, with *both* causing a low degree of freedom and/or a low value of work. It addresses the responsibility and significance of *both actor strategies* in constructing *exploitative* or *greedy relationships*. This is an important finding, since we offer some nuance to the sometimes sharp divide addressing the responsibilities of employers *or* employees in addressing the ‘shadows’ or ‘side effects’ of free movement (Mikl-Leitner, Friedrich, Teeven, & May, 2013, Asscher & Goodhart, 2013). Moreover, we show a more *mutual perspective* in discussing the burdens of the European Internal Market. As such, this study shows that there are always two sides of the coin and demonstrates how both capital *and* labour, employer *and* employees, contribute significantly to an understanding of the unwanted side-effects of free movement. As such, it provides a more balanced understanding of the consequences of free movement, instead of relying on bold statements.

Secondly, while our study includes a comparative case study perspective, despite the case variance and the political-institutional differences, our data show more similarities than differences. Despite differences in transitory regimes, political composition and institutional outlook, we found striking similarities between the position of both actors in their labour markets. In Austria, the Netherlands and Sweden we observed similar cost minimisation strategies by employers next to investment or compliance strategies of employees. This absence of differences could be related to the specific case studies, but it can probably be better explained by the overall framework of Europe as an ‘Internal Market’ in which market forces and liberal strategies play upon capital and labour actors in a way that overrides national differences. It shows that despite all the particularities of our cases, that transitory regimes, political composition and institutional outlook play a minor role in influencing transnational market processes. Our comparative perspective shows in any case that the phenomena we observed are not limited to member-state borders at all. This came as a surprise and future research might want to investigate to what extent this is true in other cases as well.

Thirdly, by highlighting *exploitative* or *greedy relationships,* this study shows that the labour market position of European mobile workers, especially in low skilled positions, does not differ from significantly from that of undocumented or irregular migrants such as Third Country Nationals (Ruhs & Anderson, 2010; Bommes & Sciortino, 2011). Of course, their legal position, rights and civic status are different and they differ in the opportunity to move up the socio-economic ladder (Snel et al., 2015). As our data focuses more on low-skilled positions, we see a rather comparable picture of *low rewarded exchange values* and *minimal valuations* on human development and autonomy, especially in those sectors of the labour market where the potential of *free* movement is not fully endorsed. If we compare mobile workers and irregular migrants, there is still some work to be done, especially when it comes to protecting those in the most vulnerable positions on the labour market. As our respondents referred mostly to the low-skilled sector we observed the importance of precariousness and vulnerability, something which has striking resemblances hinting towards a ‘new Victorian servant class’ or a ‘new precariat’, characterised by a lack of agency, stability and security (Favell, 2008; Standing, 2011). As such, it can be applauded that labour migration in low-waged labour markets is increasingly a feature in more general debates about ‘precarious work’ and the ‘new age of insecurity’ while it stays rather unclear how ‘precariousness’ and ‘vulnerability’ might be studied (Sennet, 1998; Beck, 1992; Papadopoulos, Stephenson, & Tsianos, 2008; Anderson, 2010). This study conceptualised labour commodification and operationalised capital-labour relationships in such a way that concepts such as ‘precariousness’ and ‘vulnerable work’ became visible in a comparative and interrelational way, as an analytical concept without the sole aim making broad sweep political statements. This study aims to contribute to that debate with conceptual clarity and operationalised applicability for future research.

## Appendix

**Table 4:** List of respondents (anonymous) used for citations

Respondent	Affiliation
Matti	Dutch temporary labour agency
Jonas	Dutch temporary labour agency
Joachim	Dutch representative of temporary labour agencies
Berry	Dutch NGO representative
Nick	Dutch civil servant
Paul	Austrian civil servant on the local level
Martin	Austrian Public Employment Service
Barbara	Austrian Economic Chamber
Daniel	Austrian NGO representative
Dennis	Representative of The Building Workers Union ‘West section’, local level
Andreas	Representative of The Building Workers Union ‘section Stockholm- Gotland’

## Footnotes

1 A commodity is ‘an external object which through its qualities satisfies human needs of whatever kind’ (Marx, 1978/2010). Therefore, labour commodification is the economic translation of work as labour.

2 Polanyi argued that making this commodity the organizing principle of society meant ‘to subordinate the substance of society itself to the laws of the market’. He calls this a ‘commodity fiction’ (1977, p. 13).

3 From an economical point of view, exploitation is understood as an inherent process in the capitalization and commodification of labour (Marx, 1978/2010).

4 From this strand of literature, social welfare policies can be de-commodification corrections on the on-going blossoming of capitalism to ensure social protection mechanisms, civil rights and welfare state services (Esping-Andersen, 1990).

5 Studies also show the importance of ‘not knowing’ or ‘not asking too much’: Informational agency enables one to know what one does not want to know (Brinkmeijer, 2011).

6 Related to the EU enlargements of 2004 (Poland, Estonia, Lithuania, Latvia, Czech Republic, Slovakia, Hungary, and Slovenia) and 2007 (Bulgaria and Romania).

7 Sweden, the Netherlands and Austria. The selection criteria for these three countries are explained in the ‘background section’. For more information on the project see: **www.project-imagination.net**.

8 This relatively high response rate can be explained since most stakeholders on the initial list redirected us to a colleague who was more suitable. We did not count this as non-response.

9 Dagevos, J. (red.) (2011) found that 74% of their Polish respondents worked in elementary occupations; twice as many as native Dutch workers. Gijsberts and Lubbers (2013, p. 90) found that 50% of their Polish and 40% of their Bulgarian respondents worked in elementary occupations.

10 In this statement, the respondent referred to ‘a new Polish person’ as a metaphor for *any* CEE migrant.
